# Cost analysis of colorectal cancer (CRC) management in UKM Medical Centre using clinical pathway

**DOI:** 10.1186/1471-2458-12-S2-A40

**Published:** 2012-11-27

**Authors:** Natrah Mohd, Sharifa Ezat, Syed Mohamed Aljunid, Mohd Rizal Abdul Manaf, Saperi Sulong, Ismail Sagap, Muhd Azrif

**Affiliations:** 1 Department of Community Health, Universiti Kebangsaan Malaysia Medical Centre, Jalan Yaacob Latif, 56000 Kuala Lumpur, Malaysia; 2 United Nations University-International Institute for Global Health, Universiti Kebangsaan Malaysia Medical Centre, Jalan Yaacob Latif, 56000 Kuala Lumpur, Malaysia; 3 Department of Health Information, Universiti Kebangsaan Malaysia Medical Centre, Jalan Yaacob Latif, 56000 Kuala Lumpur, Malaysia

## Background

Cost analysis has become a huge concern in today’s healthcare due to the rising healthcare cost and the pressure for policy makers to make appropriate resource allocations. One of the approaches to cost calculation is by activity based costing using clinical pathway. Colorectal cancer (CRC) is fast becoming a threat to an industrialising country like Malaysia due to its rapidly rising incidence trend. Therefore, this study aims to develop a clinical pathway in managing CRC in Universiti Kebangsaan Malaysia Medical Centre (UKMMC) and to determine the cost of managing CRC according to disease stages.

## Materials and methods

Expert panel discussion involving surgeons, an oncologist, a radiologist and a public health specialist was done to develop a clinical pathway and subsequently a clinical algorithm. Cost analysis was performed using activity-based costing approach. Estimation of the capital cost was made using Clinical Cost-Modelling (CCM) software version 2.0 by UKMMC. The final cost of CRC management for a year was calculated based on providers’ perspective.

## Results

The clinical pathway developed based on stages of CRC. Management of CRC was established to be more complex as the stage of the disease becomes more severe. The mean cost of treating a case of CRC in UKMMC for a year is RM20,831.36 and it ranges from RM17,624.77-RM24,225.15. The cost of managing stage I is RM12,961.00 and it increase to RM26,455.29 for stage IV . There is a significant difference in the mean cost of CRC management between stages of CRC (Kruskal Wallis,p < 0.001). Activity based costing contributes more than half of the total cost for each stage of CRC as compared to capital cost.

## Conclusion

Cost of CRC management increase with the increasing stage of the disease. Nationwide screening programme should be implemented in order to detect early cases of CRC and subsequently save the cost of CRC management.

